# Genetic and chemical analysis of olive oil produced by Greek olive cultivars: Linking genetic profiles with fatty acid composition and phenolic stability

**DOI:** 10.1016/j.fochms.2025.100292

**Published:** 2025-08-25

**Authors:** Annia Tsolakou, Kostas Ioannidis, Sofia Lymperopoulou, Panagiotis Diamantakos, Georgios Kostelenos, Eleni Melliou, Prokopios Magiatis

**Affiliations:** a Laboratory of Pharmacognosy and Natural Products Chemistry, Department of Pharmacy, National and Kapodistrian University of Athens, Panepistimiopolis Zografou, Athens 15771, Greece; b Laboratory of Sylviculture, Forest Genetics and Biotechnology, Institute of Mediterranean and Forest Ecosystems, Hellenic Agricultural Organization “Demeter”, Ilissia, 11528 Athens, Greece; c Kostelenos Olive Nurseries, 18020, Poros-Trizinias, Greece

**Keywords:** EVOO, Oxidative stability, Genetic diversity, Cultivar selection, SSR markers, ^1^H NMR spectroscopy, Oleocanthal, Oleacein, Oleocanthal (p-HPEA-EDA) (PubChem CID: 16681728), Oleacein (3,4-DHPEA-EDA) (PubChem CID: 18684078)

## Abstract

This pilot study explores the relationship between the genetic profiles of olive cultivars and monounsaturated fatty acid (MUFA) content of their oils, with emphasis on oxidative stability and phenolic integrity. Our working hypothesis was that cultivar-specific genetic variation in MUFA content, directly affects the oxidative stability of key phenolics, particularly oleocanthal and oleacein. To examine the association between genetic clustering and oleic acid content, eighty Greek olive cultivars cultivated under controlled nursery conditions were genotyped using eleven genomic simple sequence repeat (SSR) markers, and their fatty acid composition was determined by ^1^H NMR. Phenolic stability was tested using oils with contrasting MUFA levels. Genetic analysis identified three clusters. Chemical cluster analysis, by dividing cultivars into three MUFA classes, revealed significant differences among divisions. Linking genetic and lipid profile groups highlighted notable overlap. This study reveals a clear experimental association between MUFA abundance in the olive matrix and its capacity to preserve phenolic integrity. By confirming the role of MUFA content in phenolic stability, our results provide a baseline reference for early-stage cultivar selection and for future breeding programs targeting enhanced olive oil quality.

## Highlights

• SSR genotyping revealed three genetic clusters among 80 Greek olive cultivars.
• PCA reaffirmed that MUFA is the dominant factor differentiating fatty acid profiles.
• Distinct genetic cluster was distinguished by significantly elevated MUFA content.
• High MUFA content delayed oxidative degradation of oleocanthal and oleacein.

## 1 Introduction

Olive oil is characterized by a distinctive composition, combining a high content of monounsaturated fatty acids (MUFA) with a unique array of phenolic compounds. This chemical profile contributes both to its nutritional quality and to its resistance against oxidation. MUFAs are known for their beneficial effects on heart health, and their concentration in olive oil is a critical quality parameter. Other components include polyunsaturated fatty acids (PUFA) and saturated fatty acids (SFA), each contributing to the oil's properties. Along with Vitamin E and phenolic compounds, predominantly secoiridoids derived from tyrosol and hydroxytyrosol, olive oil's composition is not only complex, but also directly linked to its health-promoting properties. Well known factors that determine the quality and composition of an EVOO sample, batch or harvest season production include cultivar, growing conditions, processing methods (milling and malaxation) and storage conditions. Accordingly, different olive cultivars produce oils with varying proportions of fatty acids, phenolic compounds, and other minor constituents (Brenes et al., 2004; Cicerale et al., 2010; Clodoveo et al., 2014; Juliano et al., 2023; Mousavi et al., 2021). This diversity has drawn significant attention from the scientific community, leading to extensive genetic research and sequencing initiatives aimed at characterizing cultivar-specific profiles through molecular tools such as SSR markers (Abdessemed et al., 2015; Baldoni et al., 2009; Ben-Ayed et al., 2012; Las Casas et al., 2014; Sebastiani & Busconi, 2017a).

Phenolic compounds are key determinants of olive oil's stability, acting as antioxidants that guard against oxidation and rancidity, with higher levels resulting in greater oxidative resistance (Emmanouilidou et al., 2021). In parallel, fatty acid composition—particularly the ratio of oleic acid (C18:1) to linoleic acid (C18:2)—is critical, since higher oleic acid content has been consistently linked with improved oxidative stability (El Riachy et al., 2019; Montaño et al., 2016). Although total phenolic content (TPC) is important, individual phenolics exert specific and diverse effects over time, as shown by previous research from our group (Diamantakos et al., 2021). However, to the best of our knowledge, no studies have directly examined whether the fatty acid profile of the oil influences the stability of the phenolic fraction itself- a key component for the preservation of the EU- recognized health claim (EFSA Panel on Dietetic Products, 2011). By selecting cultivars rich in both oleic acid and targeted phenolics, producers can enhance oil stability and longevity.

Importantly, the relative proportions of saturated (SFA), monounsaturated (MUFA), and polyunsaturated fatty acids (PUFA) in EVOO remain consistent within each cultivar, with only minor variation introduced by environmental conditions, geographic origin, or fruit maturity. Previous studies have demonstrated that the primary determinant of lipid composition is the cultivar genotype itself, which establishes a characteristic and reproducible fatty acid profile across diverse conditions and tree ages (Bervillé & Breton, 2014; Di Lecce et al., 2020; El Riachy et al., 2019; Jukić Špika et al., 2021).

The objective of this study was to investigate the association between the genetic profiles of olive cultivars and the MUFA content of their oils, and to examine whether MUFA levels influence the oxidative stability of key phenolic compounds. Genetic differentiation was characterized by using genomic SSR markers to determine whether diversity among cultivars is associated with variations in fatty acid composition. This baseline research aimed to provide evidence supporting early-stage cultivar selection and breeding programs by focusing on desirable oil traits under controlled conditions, thereby omitting the confounding effects of growth and management practices. Our working hypothesis was that cultivar-dependent variation in MUFA percentage directly affects the capacity of EVOO to preserve phenolic integrity and, consequently, its health-related properties. To the best of our knowledge, this is the first study to address this relationship in Greek olive cultivars, providing a baseline for more extensive investigations with larger sample sets and multi-year evaluations in the future.

## 2 Materials and methods

### 2.1 Plant material

A total of 80 olive cultivars, grown under uniform nursery conditions with controlled environmental and agronomic factors, were used in all analyses. This nursery-based sampling, designed to minimize environmental effects, enabled a more precise assessment of the genetic contributions to the measured traits. All plant material was supplied by George Kostelenos nurseries (Galatas, Trizinia, Greece). Healthy leaves and corresponding fruit samples were collected within a one-month period, from early October to early November 2020. For each cultivar, 1–3 samples from different saplings were collected, depending on the nursery's availability.

### 2.2 Olive oil production

Approximately 1 kg of olive fruit from the sampled olive trees were used for oil production. Olive fruit samples were collected at the stage of maturity index 2.0–2.2, corresponding to early harvest, to ensure superior oil quality during processing. The crushing and malaxation of the olive fruit was conducted in a laboratory-scale olive mill, with a knife crusher and a malaxer operating at a temperature of 28 °C for 30 min, without any water addition, with a rotation rate of 45 rpm. All 80 samples were processed within 72 h since harvesting using the same protocol and the olive oil produced was stored at −20 °C, until further analysis. For each cultivar, 1–3 independent fruit samples were collected and processed separately, i.e. three independent oil extractions, depending on nursery availability.

### 2.3 Fatty acid composition and variability

Fatty acid composition was determined using ^1^H NMR spectroscopy, following the methodology described by Guillén and Ruiz (Guillén & Ruiz, 2003). Approximately 0.2 g of each olive oil sample was dissolved in 500 μL of deuterated chloroform (CDCl₃, Sigma-Aldrich, St. Louis, MO, USA) and analyzed on a Bruker Avance 400 MHz NMR spectrometer (Bruker, Rheinstetten, Germany). Spectra were acquired with 32 scans, a spectral width of 16 ppm, a relaxation delay of 1 s, and an acquisition time of 1.7 s. After Fourier transformation, baseline and phase corrections were performed using TopSpin software (Bruker BioSpin, Rheinstetten, Germany). Integration was applied to the characteristic signals at 0.83–0.93, 0.93–1.03, 1.94–2.14, 2.23–2.36, and 2.70–2.84 ppm, corresponding to terminal methyl protons, linolenic methyl protons, allylic and bis-allylic methylenes, and methylenes adjacent to carboxyl groups, respectively. Determination of the proportions of oleic (O), linoleic (L), linolenic (Ln) and saturated (S) acyl groups was carried out using the equations provided in the original study. The resulting fatty acids for each sample were assigned as follows: MUFA (monounsaturated fatty acids) represented by oleic acid in olive oil, PUFA (polyunsaturated fatty acids) as the sum of linoleic and linolenic acids, and SFA (saturated fatty acids). Detailed percentages of MUFA, PUFA, and SFA for each individual sample are available in **Supplementary Table 1**. The relative proportions of MUFA, PUFA, and SFA were used as the primary lipid composition traits for statistical comparisons among cultivars and population clusters. These three variables were the basis for all subsequent statistical analyses, including PCA, HCA, and ANOVA.

### 2.4 DNA isolation and quantification

Genomic DNA was extracted from 100 mg powdered fresh olive leaf samples, grounded to fine powder in the presence of liquid nitrogen, using the NucleoSpin Plant II extraction kit (Macherey-Nagel, Düren, Germany), following the manufacturer's instructions. The purified total DNA of the samples, before being stored at −20 °C, was assessed for both quantity and purity using the Q5000 UV–Vis spectrophotometer (Quawell, San Jose, CA, USA).

### 2.5 Simple sequence repeat (SSR) markers

Eleven nuclear microsatellite markers (Table 1) were carefully selected from existing literature for their high polymorphism, reliable and easily scorable patterns, strong discrimination power, and their demonstrated suitability in the characterization and identification of *Olea europaea* varieties (Alba et al., 2009; Baldoni et al., 2009; Belaj et al., 2012; Ben Ayed et al., 2016).

**Table 1:** SSR markers used for genotyping *Olea europaea*.

Locus	Repeat Motif	Primer Sequences 5′-3′		Expected Allele Range (bp)
		Forward	Reverse	
DCA3	(GA)_19_	CCCAAGCGGAGGTGTATATTGTTAC	TGCTTTTGTCGTGTTTGAGATGTTG	216–256
DCA5	(GA)_15_	AACAAATCCCATACGAACTGCC	CGTGTTGCTGTGAAGAAAATCG	194–214
DCA14	(CA)_18_A_6_(TAA)_7_	AATTTTTTAATGCACTATAATTTAC	TTGAGGTCTCTATATCTCCCAGGGG	145–188
DCA16	(GT)_13_(GA)_29_	TTAGGTGGGATTCTGTAGATGGTTG	TTTTAGGTGAGTTCATAGAATTAGC	121–188
DCA18	(CA)_4_CT(CA)_3_(GA)_19_	AAGAAAGAAAAAGGCAGAATTAAGC	GTTTTCGTCTCTCTACATAAGTGAC	154–201
DCA9	(GA)_23_	AATCAAAGTCTTCCTTCTCATTTCG	GATCCTTCCAAAAGTATAACCTCTC	158–208
EMO90	(CA)_10_	CATCCGGATTTCTTGCTTTT	AGCGAATGTAGCTTTGCATGT	180–197
GAPU71B	GA(AG)_6_(AAG)_8_	GATCAAAGGAAGAAGGGGATAAA	ACAACAAATCCGTACGCTTG	114–150
GAPU101	(GA)_8_(G)_3_(AG) _3_	CATGAAAGGAGGGGGACATA	GGCACTTGTTGTGCAGATTG	168–222
GAPU103A	(TC)_26_	TGAATTTAACTTTAAACCCACACA	GCATCGCTCGATTTTTATCC	120–196
UDO99–043	(GT)_12_	TCGGCTTTACAACCCATTTC	TGCCAATTATGGGGCTAACT	142–222

This table lists the simple sequence repeat (SSR) markers employed in the genetic analysis of *Olea europaea* samples, including the locus, repeat motif, forward and reverse primer sequences, and expected allele size range (bp). SSR, simple sequence repeat.

### 2.6 Polymerase chain reaction

The PCR reactions were performed in a total volume of 20 μL comprising 30 ng genomic DNA matrix. The final concentrations of reaction mix components were 1xPCR buffer (10×) (Nippon Genetics, Tokyo, Japan), 4 mM of MgCl_2_ (50 mM) (Nippon Genetics, Tokyo, Japan), including the amount of MgCl_2_ contained in PCR buffer, 1 U Fast Gene Taq DNA polymerase (5 UμL^−1^, Nippon Genetics, Tokyo, Japan), 250 μM each of dNTPs (10 mM) (Nippon Genetics, Tokyo, Japan), and 0.2 μM of each forward and reverse primer (Eurofins Genomics, Ebersberg, Germany).

Amplifications were carried out in a Bio Rad C1000 Touch PCR thermal cycler (Bio-Rad, Hercules, CA, USA), programmed at 95 ^°^C for 5 min for initial denaturation. The annealing steps and number of cycles adhered to the literature (Table 2) (Carriero et al., 2002; Cipriani et al., 2002; De La Rosa et al., 2004; Sefc et al., 2000). All samples were analyzed twice. Every set of PCR reactions included one negative and one positive control, and the amplification was verified with 1.5 % agarose gel electrophoresis.

**Table 2:** PCR conditions for SSR marker amplification.

SSR	Annealing		Number of Cycles	Source
	Temperature	Time		
DCA3, DCA5, DCA14, DCA16, DCA18	50 °C	30 s	35	Sefc et al., 2000
DCA9, EMO90	55 °C	30 s	35	De La Rosa et al., 2004
GAPU71B, GAPU101, GAPU103A	57 °C	45 s	30	Carriero et al., 2002
UDO99–043	57 °C	45 s	35	Cipriani et al., 2002

PCR conditions, including annealing temperatures, times, and cycle numbers, for each SSR marker group. SSR, simple sequence repeat.

### 2.7 Fragment analysis

Fragment analysis, separation, and detection of PCR products were performed on the 3730 Genetic Analyzer (Applied Biosystems, Thermo Fisher Scientific Co., Waltham, MA, USA). An aliquot (1 μL) of PCR product was added to 9 μL of cocktail, i.e., 8.5 μL Hi-Di Forma-mide® and 0.5 μL LIZ® 500 Size Standard (Applied Biosystems, Thermo Fisher Scientific Co., Waltham, MA, USA). Samples were then denatured for 3 min at 95 °C and immediately chilled on ice for 2 min and loaded on the 3730 Genetic Analyzer (Applied Biosystems, Thermo Fisher Scientific Co., Waltham, MA, USA) and run using the following conditions: oven at 63 °C; pre-run 15 kV, 180 s; injection 1.6 kV, 5 s; run 15 kV, 1600 s; capillary length 50 cm; polymer: POP-7™; and dye set G5. A customized bin set was designed, and an allelic ladder (generated from sequence data for each marker) was included with each injection to ensure accurate genotyping. Genotyping was performed using Geneious Prime v. 2022.1.1 software (Dotmatics, Boston, MA, USA). The analytical threshold was set at 500 relative fluorescence units (RFUs).

### 2.8 Genetic analysis

The number of observed alleles per locus (No), effective number of alleles (Ne), the observed and expected heterozygosity (Ho and He) were calculated using GenAlEx v.6.501 (Peakall & Smouse, 2012). The frequency of null alleles (Fnull) and Polymorphism Information Content (PIC) were estimated using Cervus v.3.0.7 (Botstein et al., 1980; Kalinowski et al., 2007). To assess the genetic structure and determine the optimal number of genetic clusters K, STRUCTURE 2.3.4 software (Pritchard et al., 2000), a Bayesian-based clustering method, was applied. For each K, ranging from 1 to 10, ten independent runs were performed. Each run consisted of a burn-in period of 100,000 steps and 1,000,000 Monte Carlo Markov Chain (MCMC) iterations, assuming an admixture model and correlated allele frequencies. The optimal K value was determined using the ΔK method of Evanno et al. (2005), implemented in STRUCTURE HARVESTER v.0.6.93 (Earl & vonHoldt, 2012; Evanno et al., 2005).

### 2.9 Chemotype–genotype association analysis

Further statistical analysis was conducted to assess the relation between fatty acid profiles grouping and the genetic clustering of cultivars. Hierarchical Clustering Analysis was performed based on the un-weighted pair group method with an arithmetic mean (UPGMA) algorithm. Frequency-based genetic distances were calculated, and 500 bootstrap repetitions were performed to construct an unweighted neighbor-joining dendrogram using DARWIN v 6.0.010 (Perrier & Jacquemoud-Collet, 2006) and FigTree v1.4.3 software was implemented for visualization (Rambaut & Drummond, 2010).

To explore the potential relationship between the distribution of fatty acids and genetic profiles of olive cultivars, a factor analysis was first performed using SPSS, V. 29 (IBM Corp., 2022, Armonk, NY, USA). Principal Component Analysis (PCA) was employed to determine the main components explaining the variance in fatty acid profiles. To enhance interpretation, each sample was assigned to one of three MUFA-based groups, based on data distribution. Specifically, cultivars with MUFA values between 72.1 and 79.5 % were classified as High MUFA (green), those with values between 66.1 and 72.0 % as Medium MUFA (blue), and those ranging from 55.4 to 66.0 % as Low MUFA (red). XLSTAT v. 2023.3.1 software (Addinsoft, Paris, France) was used to visualize the principal components derived from the PCA as well as to display the distribution of the olive cultivars based on their fatty acid profiles. Prior to this, a one-way ANOVA was performed in SPSS to assess differences in MUFA content across the identified fatty acid clusters. To visualize the linkage between the identified genetic clusters and fatty acids groups among the cultivars, the PCA results were integrated into the genetic dendrogram. In general, our analysis followed experimental designs similar to those previously employed in the field (Grasso et al., 2016; Marra et al., 2013).

### 2.10 NMR quantification of phenolic compounds in olive oil

Phenolic compounds in EVOO samples were directly quantified according to Diamantakos et al. (2021). More specifically, olive oil (5.0 g) was mixed with cyclohexane (20 mL) and acetonitrile (25 mL). The mixture was homogenized using a vortex mixer for 30 s and centrifuged at 2057 ×*g* for 5 min. A part of the acetonitrile phase (25 mL) was collected, mixed with 1.0 mL of a syringaldehyde internal standard solution (0.5 mg/mL) in acetonitrile, and evaporated under reduced pressure using a rotary evaporator (Buchi, Flawil, Switzerland). The residue of the above procedure was dissolved in CDCl_3_ (750 μL), and an accurately measured volume of the solution (550 μL) was transferred to a 5 mm NMR tube. Oleocanthal and oleacein were quantified by integrating the peaks of their aldehydic protons at 9.62 ppm and 9.64 ppm, respectively.

### 2.11 Accelerated aging of extra virgin olive oil (EVOO)

To assess whether a MUFA-rich matrix offers superior protection to phenolics compared to a PUFA-rich one, a high-phenolic extra virgin olive oil (EVOO) was subjected to an accelerated aging protocol using two different 1:1 (*w*/w) oil mixtures as model systems. The first mixture (Sample A) was prepared by combining the phenolic-rich EVOO with a phenolic-free EVOO, rich in oleic acid (83.1 %), resulting in a final MUFA content of approximately 75 %. The second mixture (Sample B) was made by combining the same phenolic-rich EVOO with sunflower seed oil, which contained 31.1 % MUFA and 58.4 % PUFA; the resulting mixture had a MUFA content of 49 %. The initial composition of both samples, including their major fatty acid content and concentrations of oleocanthal and oleacein, is presented in Table 3**.** Both samples were placed in open vials and exposed to atmospheric oxygen in an oven set at 60 °C. This setup was maintained for 14 days to simulate the long term natural oxidation if the samples were stored in a cool place at room temperature, as was described in previous work from our group (Tsolakou et al., 2018). Throughout this period, the oleocanthal and oleacein content in both samples was monitored and quantified using the previously mentioned qNMR method, with all measurements performed in triplicate. The figures presenting the ^1^H NMR spectra of Sample A and Sample B taken at different time points over a 14-day accelerated aging period at 60 °C are available in the supplementary materials (**Supplementary Fig. 1** and **Supplementary Fig. 2**).

**Table 3:** Initial composition of Samples A and B before exposure to the accelerated oxidation protocol, in terms of main fatty acids and phenolic secoiridoids (qNMR quantification).

Sample	MUFA (%)	PUFA (%)	SFA (%)	Oleocanthal (mg/kg)	Oleacein (mg/kg)
Sample A	75.0	20.0	5.0	272	144
Sample B	49.0	45.0	6.0	272	144

MUFA, monounsaturated fatty acid; PUFA, polyunsaturated fatty acids; SFA, saturated fatty acids.

## 3 Results

### 3.1 Genetic diversity and clustering

Ten of the eleven initially scored SSR loci were selected for detailed analysis based on the clarity of amplification products and the reliability of scoring data. A total of 111 different alleles occurred from PCR amplifications. Table 4 summarizes the analysis of genetic diversity for every SSR locus across the eighty olive cultivars, including allelic size range, number of alleles (Na), observed heterozygosity (Ho), expected heterozygosity (He), fixation index (Fst), estimated frequency of null alleles (Fnull) and the polymorphism information content (PIC) value. A electropherogram of the DCA05 locus, illustrating typical results for both homozygous and heterozygous samples, is provided in **Supplementary Fig. 3** of the supplementary materials. The allelic size ranges for each locus vary significantly, with DCA9 having a range of 162–208 bp and DCA18 ranging from 165 to 207 bp. The number of alleles (Na) detected across these loci averages ranges from 6 (EMO90) to 16 (DCA9), with an average of 11. The effective number of alleles (Ne), calculated across all populations for each SSR locus, ranged from 2.022 ± 0.437 (DCA5) to 6.498 ± 1.186 (DCA9). The average Ne across loci was 4.509, indicating a moderate to high level of allelic diversity in the studied germplasm. The observed heterozygosity (Ho) ranges from 0.385 (DCA5) to 0.920 (GAPU101), with an average of 0.777. DCA5 shows the lowest heterozygosity, while GAPU101 has the highest, suggesting greater diversity at this locus. Similarly, expected heterozygosity (He) values range from 0.458 (DCA5) to 0.832 (DCA9), with an average of 0.731. The high allelic count at DCA9 indicates substantial variability, while EMO90 shows lower variability. Observed heterozygosity (Ho) ranges from 0.385 at DCA5 to 0.920 at GAPU101, with an average Ho of 0.777 across all loci. Expected heterozygosity (He) is also high, with an average of 0.731. The fixation index (Fst) values range from 0.019 (GAPU71B) to 0.129 (DCA14), with an average of 0.049, indicating little genetic differentiation among loci. DCA14, with an Fst of 0.129, stands out as the locus with the highest differentiation among varieties. The mean polymorphism information content (PIC) across the ten selected loci was 0.738, confirming the high informativeness and discriminatory power of the SSR markers used.

**Table 4:** Genetic diversity across olive cultivars.

SSR locus	Allelic size range	Na	Ne	Ho	He	Fst	Fnull	PIC
DCA9	162–208	16	6.498	0.785	0.832	0.040	0.026	0.836
DCA5	192–214	9	2.022	0.385	0.458	0.041	0.050	0.477
DCA18	165–207	14	6.152	0.778	0.815	0.052	0.021	0.823
EMO90	185–199	6	3.46	0.691	0.694	0.043	0.002	0.691
GAPU101	185–218	9	4.48	0.920	0.774	0.055	0.083	0.777
GAPU71B	109–142	7	4.196	0.878	0.760	0.019	0.067	0.762
UDO99–043	160–220	15	3.43	0.747	0.667	0.040	0.048	0.680
DCA14	145–187	11	3.539	0.800	0.681	0.129	0.071	0.693
DCA3	231–253	9	5.296	0.892	0.804	0.041	0.049	0.809
DCA16	124–198	15	6.011	0.893	0.822	0.026	0.039	0.830
Αverage		11.1	4.509	0.777	0.731	0.049	0.026	0.738

Summary of allelic size range, number of alleles (Na), effective number of alleles (Ne),observed heterozygosity (Ho), expected heterozygosity (He), fixation index (Fst), estimated frequency of null alleles (Fnull) and the polymorphism information content (PIC) value for each SSR locus. SSR, simple sequence repeat.

**Fig. 1:**
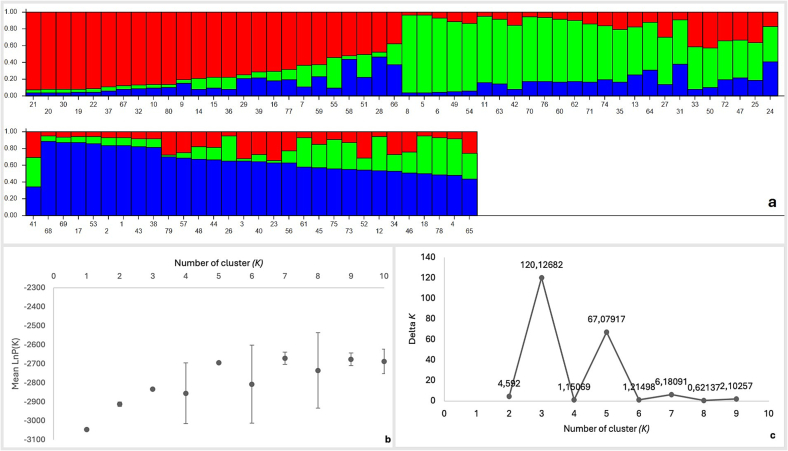
(a) Results of STRUCTURE analyses performed with K = 3, showing the proportion of the membership coefficient for each individual assigned to the inferred clusters. Each individual is represented by a vertical line partitioned into colored segments, indicating its estimated membership fraction in the three genetic clusters. (b) Plots of the ΔK value calculated according to Evanno et al. (2005), plotted against the number of modelled clusters (K). (c) Plot of the mean log-likelihood and the data [LnP(K)] ± standard deviation across STRUCTURE runs for each K value.

Genetic clustering analysis, based on Evanno's method, indicated that subdivision into three genetic clusters was the most likely scenario. The result is displayed in the bar plot in Fig. 1. Plots of the delta K value and Mean LnP(K), calculated according to Evanno et al. (2005) are also presented in Fig. 1.

### 3.2 Statistical analysis of MUFA variability

To assess the suitability of the dataset for PCA, we conducted Kaiser-Meyer-Olkin (KMO) and Bartlett's Test of Sphericity. While the KMO value was mediocre (0.690), indicating marginal sampling adequacy, Bartlett's Test yielded a significant result (*p* < 0.001), confirming that the data is appropriate for PCA. The PCA identified MUFA as the key factor of the principal component influencing fatty acid variability among the olive cultivars. The PCA correlation matrix revealed a strong negative correlation between MUFA and both PUFA (−0.881) and SFA (−0.638), indicating that as the proportion of MUFA increases, the levels of both PUFA and SFA tend to decrease. The total variance explained by the PCA indicated that two components account for more than 99 % of the total variance in the fatty acid profile, with the first component (dominated by MUFA) explaining 72.93 % of the variance, and the second component (related to PUFA) accounting for 27.06 %. This highlights MUFA as the primary factor influencing the variability in the fatty acid composition among the olive cultivars. The observed fatty acid composition in our samples displayed significant variability, with MUFA, PUFA, and SFA proportions often falling within but also extending beyond the typical ranges reported in olive oil (MUFA: 55–83 %, PUFA: 3.5–21 %, and SFA: 7.5–20 %) (Kosma et al., 2017; Revelou et al., 2021). A detailed representation of the samples' lipid profiles is provided in **Supplementary Fig. 4** of the supplementary materials.

**Fig. 2:**
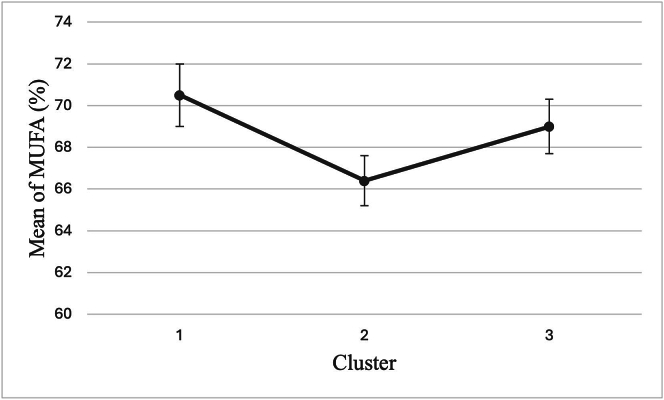
Mean MUFA levels across the three clusters.

The one-way ANOVA revealed statistically significant differences among the three aforementioned clusters (F(2,77) = 3.10, *p* = 0.049). Post hoc analysis using Tukey's test showed that Cluster 1 had significantly higher MUFA levels compared to Cluster 2 (p = 0.049). No significant differences were found between Cluster 1 and Cluster 3 (*p* = 0.771), or between Cluster 2 and Cluster 3 (*p* = 0.155). These results are visualized in Fig. 2, while the complete descriptive statistics, including confidence intervals and ranges, are available in the supplementary materials **(Supplementary Table 2)**.

### 3.3 Linking genetic structure to fatty acid patterns

**Fig. 3:**
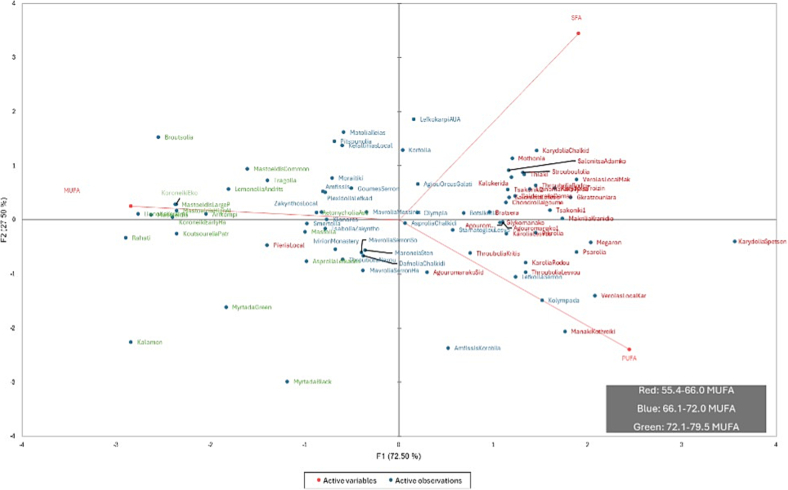
Biplot of olive cultivars based on fatty acid profiles. The biplot represents the clustering of olive cultivars based on a factor analysis of their fatty acid profiles (MUFA, PUFA, and SFA). The three fatty acid components were reduced into two factors, displayed on the axes. Samples are colour-coded according to their MUFA category: green for High MUFA (72.1–79.5 %), blue for Medium MUFA (66.1–72.0 %), and red for Low MUFA (55.4–66.0 %). Factor analysis was performed using XLSTAT (Addinsoft, v. 2023.3.1). (For interpretation of the references to colour in this figure legend, the reader is referred to the web version of this article.)

The PCA plot (Fig. 3) revealed a clear pattern: cultivars displayed continuous but structured variation along the primary axis, largely driven by MUFA content.

**Fig. 4:**
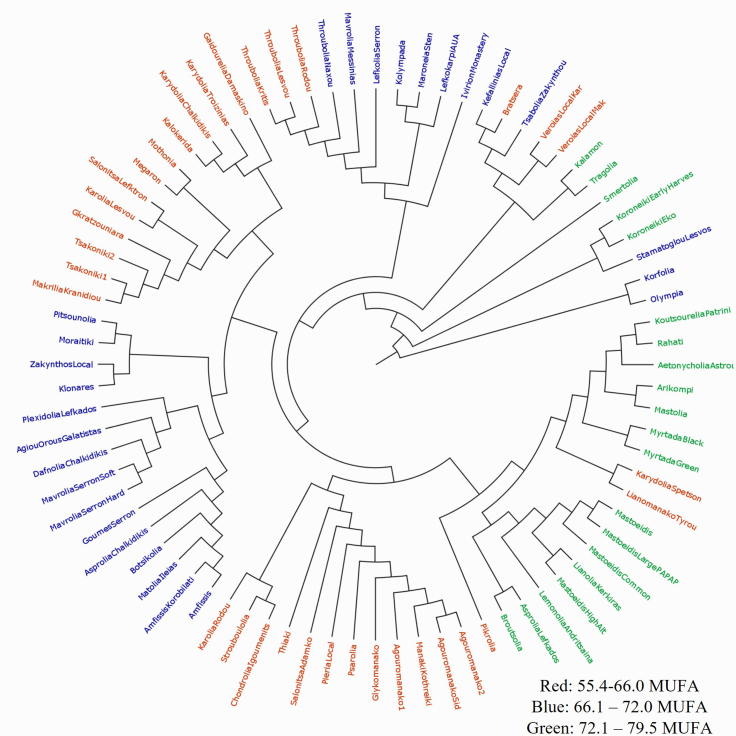
Dendrogram of olive cultivars based on ssr marker data. The dendrogram displays the hierarchical clustering of olive cultivars using data from 10 SSR markers. Each branch represents genetic relationships between cultivars, with colors corresponding to MUFA categories: green for high MUFA, blue for medium MUFA, and red for low MUFA. While smaller clusters showed alignment with MUFA levels, larger branches exhibited mixed categories, suggesting that genetic factors beyond the SSR markers analyzed may contribute to the variability in fatty acid composition. (For interpretation of the references to colour in this figure legend, the reader is referred to the web version of this article.)

Hierarchical Cluster Analysis (HCA) revealed distinct groupings of olive cultivars based on their genetic background. The alignment with MUFA categories was evident, particularly in those grouping together high MUFA (green) or low MUFA (red) cultivars (Fig. 4). However, some varieties did not consistently cluster according to their MUFA levels, indicating partial overlap. The major branches of the dendrogram included a mixture of cultivars with varying MUFA content. Given the minimization, as possible, of environmental and agronomic influences, largely controlled under nursery conditions, the observed variability in MUFA content among cultivars can, to an extent, be attributed to genetic differentiation at the varietal level.

### 3.4 Validation of MUFA-associated phenolic stability through accelerated aging

**Fig. 5:**
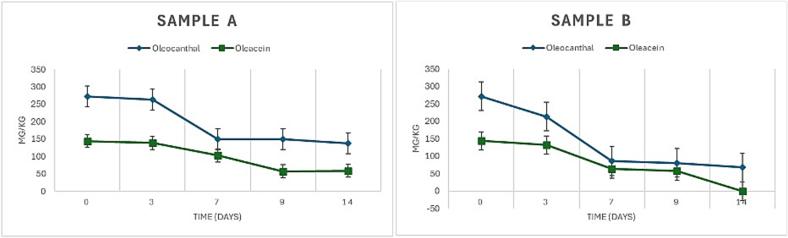
Kinetics of phenolic compounds degradation during accelerated aging at 60 °C.

The degradation kinetics of oleocanthal and oleacein were assessed under oxidative stress conditions in two oil blends with contrasting fatty acid compositions. As illustrated in Fig. 5, the phenolic content declined at a markedly different rate between the two samples during the 14-day incubation at 60 °C. In the high-MUFA blend (Sample A), oleocanthal levels decreased by approximately 40 %, and oleacein content dropped by 58 %. In contrast, the low-MUFA/high-PUFA blend (Sample B) exhibited a far more rapid degradation: oleocanthal was reduced by 80 %, and oleacein was undetectable after Day 9. Our results demonstrate that phenolic compounds degrade significantly faster in PUFA-rich environments, while a higher oleic acid content appears to offer a protective effect against oxidation-induced losses. The findings support the hypothesis that fatty acid composition directly impacts the oxidative stability of bioactive phenolic constituents in olive oil.

Time-course degradation profiles of oleocanthal (blue) and oleacein (green) over a 14-day oxidative stress test in two oil matrices. Sample A consists of high-MUFA extra virgin olive oil, while Sample B includes sunflower seed oil (high PUFA content). Phenolic concentrations (mg/kg) were determined using qNMR. The MUFA-rich environment in Sample A significantly delayed the degradation of both phenolic compounds compared to the PUFA-rich environment of Sample B, illustrating the protective role of fatty acid composition on phenolic stability.

## 4 Discussion

### 4.1 Contribution to the existing literature

Previous studies have examined the relationship between the genetic profile of olive trees and the chemical composition of their oil, often focusing on specific varieties or origins (Dabbou et al., 2012; Jukić Špika et al., 2021). Understanding these relationships can support producers in selecting appropriate cultivars, aid in the development of targeted breeding strategies and enhance the production of high-quality olive oil. The most abundant MUFA in olive oil is oleic acid (C18:1), which contributes to its characteristic flavor and stability. PUFAs, including linoleic acid (C18:2) and linolenic acid (C18:3), are present in lower amounts, while SFAs, such as palmitic acid (C16:0), are less prevalent in olive oil. The genetic makeup of olive trees plays a crucial role in determining the composition of the produced EVOO (Emmanouilidou et al., 2021; Ipek et al., 2015; Sebastiani & Busconi, 2017b). The cultivar-dependent nature of phenolic composition has been confirmed through an extensive germplasm survey, highlighting the importance of genetic factors in olive oil quality (Tomé-Rodríguez et al., 2023a). Moreover, several studies have demonstrated how cultivar genetic profiles influence olive oil characteristics, particularly under optimized irrigation, nutrient management, and harvest timing conditions (Contreras et al., 2023; Hernández et al., 2021; Omri et al., 2021; Tomé-Rodríguez et al., 2023b). Some cultivars naturally produce higher levels of oleic acid, resulting in extra virgin olive oils with excellent taste and stability, while others may have higher linoleic acid content, affecting oil quality. In parallel, the genetic diversity and relationships among Greek olive cultivars have been extensively studied using molecular markers, contributing to a better understanding of their population structure and evolutionary background (Linos et al., 2014).

Dabbou et al. (2012) using principal component analysis (PCA) has classified olive oil hybrids based on their fatty acid profiles and identified correlations between specific fatty acids (Dabbou et al., 2012). Our own data support this observation, indicating that oleic acid (C18:1) and linoleic acid (C18:2) tend to be inversely related. As depicted by other articles, the expression levels of fatty acid desaturase genes (OeFAD2–2 and OeFAD2–5) and the specificities of extraplastidial acyltransferase enzymes contribute to the variability of the oleic/linoleic acid ratio in olive cultivars, advancing our understanding of linoleic acid biosynthesis and potentially enhancing olive breeding programs for improved oil quality (Hernández et al., 2021). Olive oil producers can select cultivars based on desired fatty acid profiles, thereby offering consumers products with enhanced health benefits and superior organoleptic qualities. High oleic acid content contributes to stability and health benefits and simultaneously low linoleic acid content ensures oxidative resistance (Velasco & Dobarganes, 2002; Zhou et al., 2024), turning the production of high MUFA EVOO an objective for breeding programs and storage recommendations. Furthermore, phenolic compounds, which are also influenced by genetic background, play a pivotal role in enhancing the antioxidant properties and health benefits of olive oil.

In our study, we identified three distinct MUFA categories—high, medium, and low—based on the fatty acid profiles of the oils produced from 80 different cultivars. While we initially hypothesized a strong association between the genetic profile of the studied varieties and their MUFA content, our findings revealed non-exclusive but consistent trends. A general grouping trend was observed, supporting the widely accepted view that genotype is considered to be the major source of variability for VOO fatty acid composition (Ripa et al., 2008; M. Torres et al., 2017). It is worth noting that 85 % of the high-MUFA cultivars fall into the small-fruited category (Supplementary Table 1), which often aligns with their traditional use for oil production — possibly reflecting long-standing human-driven selection for processing suitability. In addition, the stability of phenolic compounds, specifically oleocanthal and oleacein, was significantly influenced by the MUFA content of the oils. In the high-MUFA environment, phenolic degradation occurred at a much slower rate compared to the PUFA-rich oils, supporting the hypothesis that MUFA content is a key factor in maintaining phenolic stability. These results align with previous studies that have demonstrated the stabilizing effect of oleic acid on phenolic compounds (Kosma et al., 2017), further confirming the importance of MUFA-rich cultivars for producing olive oil with enhanced oxidative stability.

Our study holds significant value as a baseline survey establishing a crucial link between genetic structure and fatty acid variation in olives, with a foundational contribution regarding Greek accessions. It provides the first comprehensive dataset correlating olive genetic diversity with fatty acid profile variation, serving as a critical reference for future studies. The observed FA–genetic correlations have direct applications in olive oil quality improvement and early-stage cultivar selection.

In Greece, approximately 100 cultivars have been officially recognized; however, only 20–30 are actively cultivated, while the remainder are not exploited commercially. Given that the total agricultural area of the country is relatively limited compared with other major olive-producing nations such as Spain and Italy, the diversity of cultivars does not constitute a substantial constraint. The present study encompassed more than 80 % of the recognized Greek cultivars, including all cultivars that are extensively and commercially cultivated within the country. Our methodological approach also followed similar designs previously employed in the field (Grasso et al., 2016; Marra et al., 2013).

### 4.2 Study design and scope

One of the primary strengths of this study was the effort to separate the genetic background underlying phenotypic variability, by reducing environmental influences. This was achieved by cultivating all olive trees under uniform environmental and agronomic conditions in a nursery, thereby reducing the environmental component which affects the traits studied. Moreover, by applying standardized laboratory processing methods across all samples, we were able to emphasize the genetic component influencing the oil's properties. Genotype is considered the primary source of variability in the fatty acid composition of virgin olive oil (Ripa et al., 2008), but environmental factors and the genotype × environment interaction can also have significant effects (Ceci & Carelli, 2007; Rondanini, Castro, Searles and Rousseaux, 2011, Rondanini, Castro, Searles and Rousseaux, 2014a; M. Torres et al., 2017; M. M. Torres et al., 2009; M. M. Torres & Maestri, 2006). In this context, our approach gains particular significance as previous studies have shown that certain European olive cultivars grown in various regions of South America and Australia produce oils with fatty acid compositions that differ significantly from those of the same cultivars grown in their native Mediterranean Basin (Bodoira et al., 2016; Ceci & Carelli, 2007; Mailer et al., 2010; Rondanini, Castro, Searles and Rousseaux, 2011, Rondanini, Castro, Searles and Rousseaux, 2014b; M. Torres et al., 2017; M. M. Torres et al., 2009; M. M. Torres & Maestri, 2006).^.^ This occurs despite adherence to proper agronomic practices, harvesting methods, and fruit processing standards (M. Torres et al., 2017). It should be noted, however, that fatty acid composition in olive oil is also known to be influenced by fruit ripening (Amanpour et al., 2019), climatic parameters, growing area conditions, rainfall, and temperature during the oil accumulation phase (García-Inza et al., 2014; Issaoui et al., 2010; Rizwan et al., 2019). In our study, the experimental design intentionally excluded such climatic influences and genotype × environment interactions, as all accessions were grown under uniform nursery conditions with controlled environmental and agronomic factors. By minimizing environmental variation, the genetic effects on phenotypic traits could be more reliably estimated, and interannual variability in oil composition was expected to be minimal. Previous studies further support that (a) efficient selection for fatty acid profiles can be achieved through single-year phenotypic evaluation at the seedling stage (León et al., 2008), and (b) only some cultivars show a genotype × environment effect on oleic acid variability (Rondanini et al., 2011). Hence, our methodology allowed us to appropriately examine the association between genetic variation and both fatty acid composition and phenolic stability.

### 4.3 Practical applications and cultivar improvement

Our findings highlight the importance of MUFA content in preserving the phenolic stability and maintaining health-claim eligibility of extra virgin olive oil. Cultivars with higher levels of oleic acid demonstrated greater oxidative stability and a slower rate of phenolic compounds degradation, particularly in compounds such as oleocanthal and oleacein, which are critical for both the health benefits and shelf life of olive oil. These results suggest that producers should be cautious when blending oils from different cultivars, as mixing high-MUFA oils with those containing lower MUFA content could compromise the phenolic stability and oxidative resistance of the final product.

### 4.4 Limitations

While our study provides novel insights into the association between genetic structure and fatty acid composition in Greek olive cultivars, certain limitations should be acknowledged. Both the number of genotypes analyzed (80 cultivars) and the number of SSR loci employed (eleven) represent a restricted dataset for association analyses. Nevertheless, our approach was guided by comparable studies in the field that have successfully applied similar experimental designs (Grasso et al., 2016; Marra et al., 2013). These studies demonstrated that even with relatively modest marker panels and sample sizes, meaningful patterns of genetic–chemical associations can be revealed. Thus, the current work can be considered as a justified baseline contribution that can support future studies with expanded germplasm collections and more extensive marker sets.

## 5 Conclusions

Although genetic variation is associated with fatty acid composition, our results suggest that the observed intra-cluster variability points to opportunities for further improvement through targeted breeding strategies and the deliberate selection of cultivars to enhance olive oil quality. This variability can be strategically exploited by olive oil producers through the selective cultivation of varieties with naturally higher MUFA content, particularly those rich in oleic acid. Moreover, targeted agronomic practices, such as optimized irrigation, nutrient management, and harvest timing, may further increase the MUFA percentage in olive oil. Studies on how genetic profiles influence the predisposed characteristics of olive oil, especially in response to the above particular conditions, can guide future strategies for olive cultivation and varietal selection. By choosing cultivars based on their genetic predisposition for favorable fatty acid profiles in parallel with refining cultivation techniques, producers can enhance both the quality and health benefits of the oil. The ultimate goal is to not only exploit the genetic potential of each variety but also to support a model of olive oil production that combines bioactive potency with high added value across nutritional and commercial dimensions.

## CRediT authorship contribution statement

**Annia Tsolakou:** Writing – original draft, Investigation, Formal analysis, Data curation. **Kostas Ioannidis:** Writing – review & editing, Writing – original draft, Resources, Methodology, Investigation. **Sofia Lymperopoulou:** Formal analysis, Data curation. **Panagiotis Diamantakos:** Investigation, Formal analysis, Data curation. **Georgios Kostelenos:** Resources, Methodology. **Eleni Melliou:** Writing – review & editing, Methodology, Funding acquisition, Conceptualization. **Prokopios Magiatis:** Writing – review & editing, Supervision, Resources, Project administration, Methodology, Conceptualization.

## Declaration of competing interest

The authors declare the following financial interests/personal relationships which may be considered as potential competing interests: Ioannidis Kostas reports equipment, drugs, or supplies was provided by World Olive center for health. Melliou Eleni reports a relationship with World Olive center for health (non profit organization) that includes: board membership. If there are other authors, they declare that they have no known competing financial interests or personal relationships that could have appeared to influence the work reported in this paper.

## Data Availability

Data will be made available on request.

## Appendix A Supplementary data

Supplementary material with genetic population assignments, fatty acid composition, MUFA categorization, and fruit size of the olive cultivars studied. Descriptive statistics by genetic cluster, representative ¹H NMR spectra of phenolic degradation in contrasting MUFA/PUFA oils, chromatograms of SSR marker genotyping, and bar plots of fatty acid profiles are also provided. Image 1
