# Supplementary material for: Genetic and chemical analysis of olive oil produced by Greek olive cultivars: Linking genetic profiles with fatty acid composition and phenolic stability
Source: Food Chem (Oxf). 2025 Aug 25;11:100292. doi: 10.1016/j.fochms.2025.100292 (PMC12433495; doi:10.1016/j.fochms.2025.100292)
Supplement: Supplementary file 1 — Supplementary material with genetic population assignments, fatty acid composition, MUFA categorization, and fruit size of the olive cultivars studied. Descriptive statistics by genetic cluster, representative ¹H NMR spectra of phenolic degradation in contrasting MUFA/PUFA oils, chromatograms of SSR marker genotyping, and bar plots of fatty acid profiles are also provided. [file mmc1.docx]

**Genetic and Chemical Analysis of Olive Oil produced by Greek Olive Cultivars: Linking Genetic Profiles with Fatty Acid Composition and Phenolic Stability**

Annia Tsolakou^a^, Kostas Ioannidis^b,*^, Lymperopoulou Sofia^a^, Panagiotis Diamantakos^a^, Georgios Kostelenos^c^, Eleni Melliou^a^ and Prokopios Magiatis^a,*^

^a^ Department of Pharmacognosy and Natural Products Chemistry, Faculty of Pharmacy, National and Kapodistrian University of Athens, Panepistimiopolis Zografou, Athens 15771, Greece; [atsolakou@pharm.uoa.gr](mailto:atsolakou@pharm.uoa.gr) (A.T.); [pdiam@pharm.uoa.gr](mailto:pdiam@pharm.uoa.gr) (P.D.); [emelliou@pharm.uoa.gr](mailto:emelliou@pharm.uoa.gr) (E.M.); [magiatis@pharm.uoa.gr](mailto:magiatis@pharm.uoa.gr) (P.M.)

^b^ Laboratory of Sylviculture, Forest Genetics and Biotechnology, Institute of Mediterranean and Forest Ecosystems, Hellenic Agricultural Organization “Demeter”, Ilissia, 11528 Athens, Greece; [ioko@fria.gr](mailto:ioko@fria.gr) (K.I.)

^c^ Kostelenos Olive Nurseries, 18020, Poros-Trizinias, Greece

*Correspondence. [magiatis@pharm.uoa.gr](mailto:magiatis@pharm.uoa.gr); Tel.: +30 210 7274052 (P.M).

*Correspondence: [ioko@fria.gr](mailto:ioko@fria.gr); Tel.: +30 210 7783750 (I.K).

# **Supplementary Material**

**Supplementary Table 1.** Olive cultivars, genetic populations, fatty acid composition, MUFA content categorization and fruit size.

| Cultivar | Population | MUFA(%) | PUFA(%) | SFA(%) | Category | Fruit size |
| --- | --- | --- | --- | --- | --- | --- |
| Rahati | 1 | 79.5 ± 0.9 | 5.1 ± 0.2 | 15.4 ± 0.5 | green | 1 |
| Lianolia Kerkiras | 1 | 79.1 ± 3.6 | 4.4 ± 0.2 | 16.5 ± 0.5 | green | 1 |
| Broutsolia | 1 | 78.8 ± 1.4 | 1.4 ± 0.0 | 19.8 ± 0.3 | green | 1 |
| Kalamon | 3 | 78.6 ± 2.9 | 10.1 ± 0.2 | 11.3 ± 0.1 | green | 3 |
| Mastoeidis | 1 | 78.6 ± 2.0 | 4.8 ± 0.1 | 16.6 ± 0.7 | green | 1 |
| Koroneiki EH | 3 | 77.7 ± 2.6 | 5.4 ± 0.2 | 16.8 ± 0.5 | green | 1 |
| Koroneiki | 3 | 77.7 ± 3.1 | 4.9 ± 0.2 | 17.4 ± 0.7 | green | 1 |
| Mastoeidis LF | 1 | 77.7 ± 3.0 | 4.9 ± 0.2 | 17.4 ± 0.3 | green | 1 |
| Mastoeidis HA | 1 | 77.6 ± 3.2 | 5.3 ± 0.1 | 17.1 ± 0.7 | green | 1 |
| Koutsourelia Patras | 1 | 77.5 ± 1.9 | 6.3 ± 0.1 | 16.2 ± 0.7 | green | 1 |
| Arikompi | 3 | 76.4 ± 1.4 | 6.2 ± 0.3 | 17.4 ± 0.2 | green | 1 |
| Lemonolia Andritsainas | 3 | 75.7 ± 2.4 | 5.7 ± 0.1 | 18.6 ± 0.3 | green | 1 |
| Mastoeidis C | 1 | 75.1 ± 3.2 | 5.2 ± 0.1 | 19.7 ± 0.9 | green | 1 |
| Myrtada G | 3 | 75.1 ± 1.3 | 11.0 ± 0.5 | 13.9 ± 0.1 | green | 1 |
| Tragolia | 3 | 74.2 ± 0.5 | 6.3 ± 0.1 | 19.5 ± 0.3 | green | 1 |
| Mastolia | 2 | 72.4 ± 1.2 | 9.7 ± 0.3 | 17.9 ± 0.4 | green | 1 |
| Smertolia | 1 | 72.4 ± 2.2 | 9.4 ± 0.3 | 18.3 ± 0.6 | green | 1 |
| Asprolia Lefkados | 1 | 72.2 ± 2.4 | 11.1 ± 0.4 | 16.8 ± 0.4 | green | 2 |
| Myrtada B | 3 | 72.2 ± 2.6 | 16.1 ± 0.6 | 11.7 ± 0.4 | green | 1 |
| Aetonycholia Astrous | 1 | 72.1 ± 0.8 | 9.1 ± 0.2 | 18.8 ± 0.2 | green | 3 |
| Plexidolia Lefkados | 3 | 72.0 ± 2.4 | 8.3 ± 0.3 | 19.7 ± 0.8 | blue | 1 |
| Amfissis | 2 | 71.9 ± 2.8 | 8.4 ± 0.4 | 19.7 ± 0.3 | blue | 3 |
| Zakynthos Local | 1 | 71.9 ± 1.9 | 9.2 ± 0.2 | 18.9 ± 0.8 | blue | 1 |
| Pitsounolia | 3 | 71.8 ± 1.9 | 6.3 ± 0.2 | 21.9 ± 0.4 | blue | 1 |
| Moraitiki | 3 | 71.7 ± 3.1 | 7.9 ± 0.3 | 20.4 ± 0.3 | blue | 2 |
| Klonares | 3 | 71.6 ± 2.0 | 9.7 ± 0.3 | 18.7 ± 0.5 | blue | 2 |
| Tsabolia Zakynthou | 3 | 71.6 ± 3.0 | 10.1 ± 0.4 | 18.3 ± 0.8 | blue | 1 |
| Kefallinias Local | 3 | 71.5 ± 2.4 | 6.7 ± 0.3 | 21.8 ± 0.9 | blue | 1 |
| Matolia Ileias | 3 | 71.5 ± 2.8 | 6.1 ± 0.3 | 22.4 ± 1.0 | blue | 2 |
| Ivirion Monastery | 3 | 71.1 ± 3.1 | 11.3 ± 0.3 | 17.6 ± 0.1 | blue | 1 |
| Goumes Serron | 2 | 70.8 ± 2.6 | 8.9 ± 0.2 | 20.2 ± 0.4 | blue | 2 |
| Throubolia Naxou | 1 | 70.8 ± 1.8 | 12.0 ± 0.4 | 17.3 ± 0.3 | blue | 2 |
| Mavrolia Messinias | 3 | 70.1 ± 2.1 | 10.4 ± 0.2 | 19.5 ± 0.8 | blue | 1 |
| Mavrolia Serron S | 2 | 70.1 ± 2.4 | 12.1 ± 0.5 | 17.8 ± 0.2 | blue | 2 |
| Dafnolia Chalkidikis | 2 | 70.0 ± 2.0 | 12.3 ± 0.5 | 17.7 ± 0.2 | blue | 3 |
| Maroneia St | 3 | 69.9 ± 3.0 | 12.2 ± 0.3 | 17.9 ± 0.7 | blue | 2 |
| Mavrolia Serron H | 2 | 69.9 ± 1.3 | 13.0 ± 0.4 | 17.1 ± 0.5 | blue | 2 |
| Korfolia | 3 | 69.1 ± 2.8 | 8.5 ± 0.2 | 22.4 ± 0.5 | blue | 1 |
| Lefkokarpi A | 3 | 68.8 ± 2.5 | 7.4 ± 0.3 | 23.8 ± 0.8 | blue | 1 |
| Asprolia Chalkidikis | 2 | 68.5 ± 3.1 | 12.0 ± 0.6 | 19.5 ± 0.6 | blue | 3 |
| Agiou Orous Galatistas | 2 | 68.3 ± 3.0 | 10.5 ± 0.4 | 21.2 ± 0.3 | blue | 2 |
| Olympia | 2 | 68.1 ± 1.5 | 11.8 ± 0.0 | 20.1 ± 0.8 | blue | 1 |
| Kolympada | 2 | 67.7 ± 1.2 | 14.1 ± 0.6 | 18.2 ± 0.5 | blue | 3 |
| Stamatoglou Lesvos | 3 | 66.6 ± 2.4 | 13.6 ± 0.5 | 19.8 ± 0.6 | blue | 2 |
| Lefkolia Serron | 3 | 66.4 ± 2.2 | 14.8 ± 0.5 | 18.8 ± 0.8 | blue | 2 |
| Amfissis Korobilati | 2 | 66.1 ± 2.1 | 18.9 ± 0.7 | 15.0 ± 0.4 | blue | 3 |
| Botsikolia | 3 | 66.1 ± 2.5 | 13.3 ± 0.4 | 20.7 ± 0.7 | blue | 1 |
| Throubolia Kritis | 1 | 65.8 ± 3.2 | 15.1 ± 0.4 | 19.1 ± 0.8 | red | 2 |
| Agouromanako 3 | 1 | 65.5 ± 2.7 | 14.8 ± 0.2 | 19.6 ± 0.4 | red | 2 |
| Bratsera | 3 | 65.3 ± 1.8 | 13.7 ± 0.5 | 21.0 ± 1.0 | red | 1 |
| Pieria Local | 2 | 65.3 ± 0.0 | 18.3 ± 0.8 | 16.4 ± 0.5 | red | 2 |
| Glykomanako | 1 | 64.8 ± 1.8 | 14.5 ± 0.6 | 20.7 ± 0.2 | red | 2 |
| Salonitsa A | 2 | 64.8 ± 3.2 | 12.3 ± 0.5 | 22.9 ± 0.4 | red | 3 |
| Agouromanako 1 | 1 | 64.7 ± 1.5 | 14.6 ± 0.3 | 20.8 ± 0.3 | red | 2 |
| Agouromanako 2 | 1 | 64.7 ± 1.0 | 14.6 ± 0.1 | 20.8 ± 0.4 | red | 2 |
| Chondrolia Igoumenitsas | 3 | 64.7 ± 2.7 | 13.7 ± 0.3 | 21.6 ± 0.2 | red | 2 |
| Mothonia | 1 | 64.7 ± 2.4 | 11.9 ± 0.3 | 23.4 ± 0.7 | red | 2 |
| Tsakoniki B | 2 | 64.7 ± 0.7 | 13.2 ± 0.4 | 22.1 ± 0.5 | red | 1 |
| Kalokerida | 2 | 64.6 ± 2.6 | 12.7 ± 0.4 | 22.7 ± 1.1 | red | 2 |
| Salonitsa L | 2 | 64.6 ± 0.4 | 13.6 ± 0.2 | 21.9 ± 1.0 | red | 3 |
| Karolia Lesvou | 2 | 64.4 ± 1.1 | 15.2 ± 0.5 | 20.4 ± 0.8 | red | 3 |
| Gaidourelia Damaskinolia | 3 | 64.3 ± 2.9 | 13.7 ± 0.6 | 22.0 ± 0.8 | red | 3 |
| Strouboulolia | 3 | 64.2 ± 0.8 | 12.8 ± 0.2 | 23.0 ± 0.8 | red | 2 |
| Thiaki | 3 | 64.1 ± 2.6 | 13.0 ± 0.2 | 23.0 ± 0.5 | red | 1 |
| Karydolia Chalkidikis | 2 | 63.8 ± 0.5 | 12.2 ± 0.4 | 24.1 ± 0.6 | red | 3 |
| Lianomanako Tyrou | 2 | 63.8 ± 1.9 | 13.8 ± 0.2 | 22.4 ± 0.9 | red | 1 |
| Throubolia Rodou | 1 | 63.6 ± 2.1 | 13.7 ± 0.2 | 22.7 ± 0.9 | red | 2 |
| Karolia Rodou | 1 | 63.5 ± 1.4 | 17.0 ± 0.4 | 19.5 ± 0.7 | red | 2 |
| Throubolia Lesvou | 1 | 63.5 ± 1.1 | 17.5 ± 0.5 | 19.1 ± 0.6 | red | 2 |
| Pikrolia | 3 | 63.3 ± 1.6 | 15.9 ± 0.5 | 20.8 ± 0.7 | red | 2 |
| Tsakoniki A | 2 | 62.9 ± 1.4 | 15.3 ± 0.7 | 21.9 ± 0.5 | red | 2 |
| Karydolia Troizinias | 2 | 62.8 ± 1.5 | 14.4 ± 0.4 | 22.8 ± 0.3 | red | 2 |
| Makrilia Kranidiou | 2 | 62.3 ± 2.4 | 16.0 ± 0.0 | 21.7 ± 0.6 | red | 2 |
| Gkratzouniara | 2 | 62.1 ± 1.5 | 15.2 ± 0.5 | 22.6 ± 0.8 | red | 3 |
| Veroias Local M | 3 | 62.0 ± 1.8 | 14.6 ± 0.4 | 23.4 ± 0.5 | red | 2 |
| Psarolia | 1 | 61.6 ± 1.9 | 17.9 ± 0.6 | 20.5 ± 0.4 | red | 3 |
| Manaki Kothreiki | 1 | 61.5 ± 2.1 | 21.3 ± 0.7 | 17.2 ± 0.5 | red | 2 |
| Megaron | 3 | 61.1 ± 2.3 | 17.8 ± 0.3 | 21.1 ± 0.4 | red | 2 |
| Veroias Local | 2 | 60.6 ± 1.6 | 20.4 ± 0.2 | 19.0 ± 0.8 | red | 2 |
| Karydolia Spetson | 3 | 55.4 ± 2.4 | 21.7 ± 0.4 | 22.9 ± 0.9 | red | 2 |
|  | | | | | |  |

This table lists the olive cultivars used in this study, sorted by their mean MUFA content, in a descending order. Genetic population is listed as determined by STRUCTURE/Harvester softwares. For each cultivar, the mean percentage of monounsaturated fatty acids (MUFA), polyunsaturated fatty acids (PUFA), saturated fatty acids (SFA) in the produced olive oil is presented, as well as the cultivar’s fruit size.

**Supplementary Table 2.** Descriptive Statistics for MUFA Content by Genetic Cluster.

| Genetic Cluster | N | Mean | Std. Deviation | Std. Error | 95% CI Lower Bound | 95% CI Upper Bound | Minimum | Maximum |
| --- | --- | --- | --- | --- | --- | --- | --- | --- |
| 1 | 24 | 70.367 | 6.4121 | 1.3089 | 67.660 | 73.075 | 61.5 | 79.5 |
| 2 | 24 | 66.607 | 4.1749 | 0.8522 | 64.844 | 68.370 | 60.6 | 76.6 |
| 3 | 32 | 69.353 | 5.5095 | 0.9740 | 67.367 | 71.340 | 55.4 | 78.6 |
| Total | 80 | 68.834 | 5.5935 | 0.6254 | 67.589 | 70.078 | 55.4 | 79.5 |

This table presents the descriptive statistics of monounsaturated fatty acid (MUFA) content across the three genetic clusters analyzed in this study. Values include the mean, standard deviation, standard error, 95% confidence intervals, and the range (minimum and maximum) for each cluster. Data was generated using SPSS software, and the detailed summary of these statistics highlights the variability in MUFA content between populations.


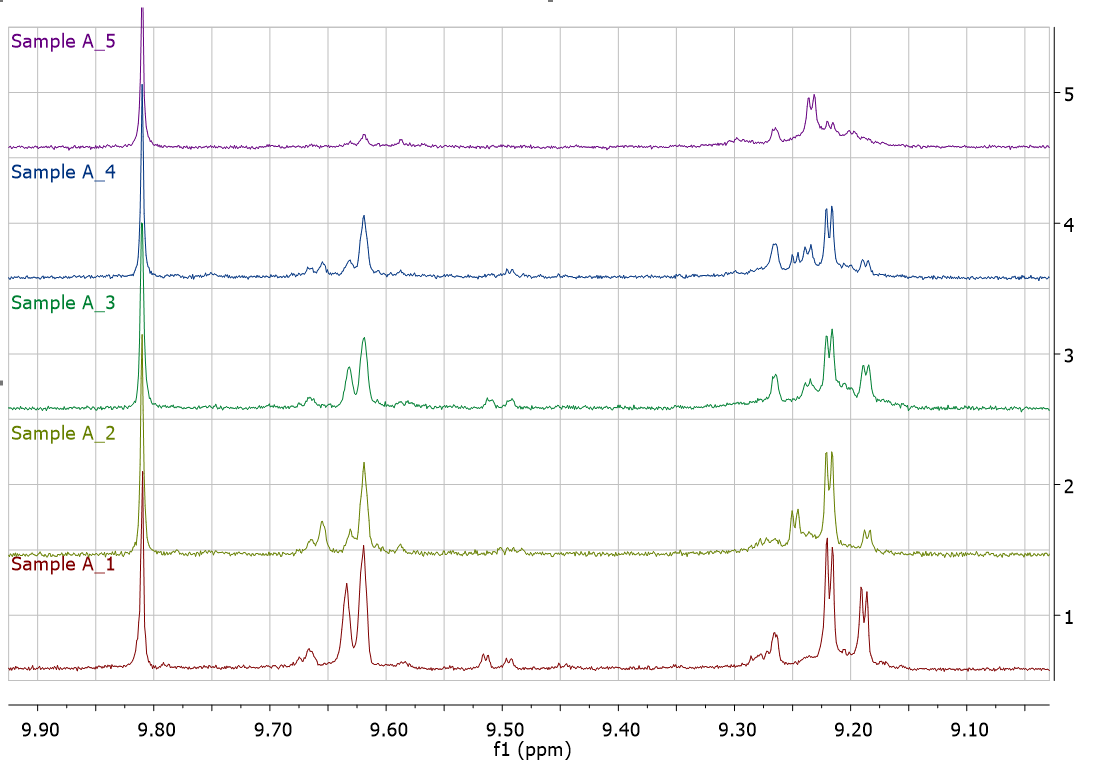
**Supplementary Figure 1. ¹H-NMR Spectra Tracking the Degradation of Oleocanthal and Oleacein in High-MUFA EVOO Blend (Sample A).**

This figure presents the ¹H-NMR spectra of Sample A (a blend with high-MUFA extra virgin olive oil) taken at different time points over a 14-day accelerated aging period at 60°C. Spectra A1 to A5 correspond to Day 0, Day 3, Day 7, Day 9, and Day 14, respectively. The peaks corresponding to oleocanthal and oleacein were monitored and compared to an internal standard (IS) peak to calculate their concentrations following the method described by Diamantakos *et al*. (2020). In this MUFA-rich environment, the slower degradation effect of high MUFA content on these phenolic compounds is apparent, in comparison to the following results of Supplementary Figure 2.

**
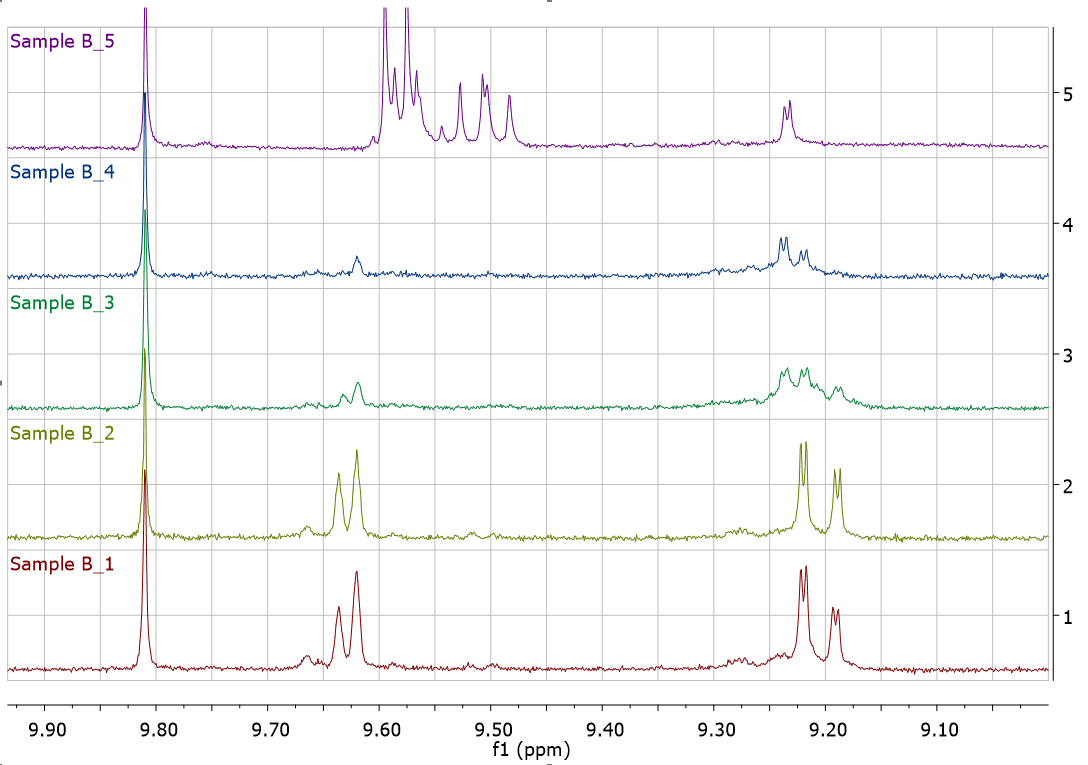
Supplementary Figure 2. ¹H-NMR Spectra Tracking the Degradation of Oleocanthal and Oleacein in Low-MUFA Sunflower Seed Oil Blend (Sample B).**

This figure displays the ¹H-NMR spectra of Sample B (a 50:50 blend with sunflower seed oil) over the same 14-day accelerated aging period at 60°C as in Sample A. Spectra B1 to B5 correspond to Day 0, Day 3, Day 7, Day 9, and Day 14. Using the method from Diamantakos *et al*. (2020), the peaks of oleocanthal and oleacein were tracked and compared to the internal standard (IS) to assess degradation. In contrast to the results from Sample A, oleocanthal in this PUFA-rich environment degraded by 80%, and oleacein was entirely degraded by Day 9, demonstrating the faster degradation of phenolic compounds in low-MUFA environments.


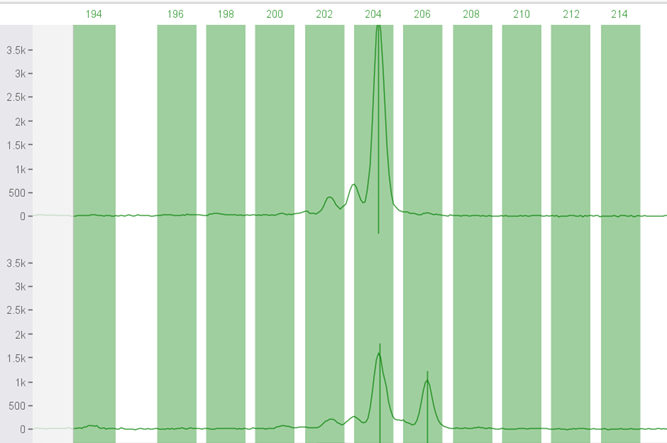


**Supplementary Figure 3.** **Chromatogram of DCA05 Amplicons Showing Typical Results for Homozygous and Heterozygous Samples**.

The figure presents chromatograms for two samples genotyped with the DCA05 SSR marker. One sample is homozygous (up), and the other is heterozygous (down), both displaying peak heights well above 500 RFU, ensuring reliable and clean results, free from artifacts.


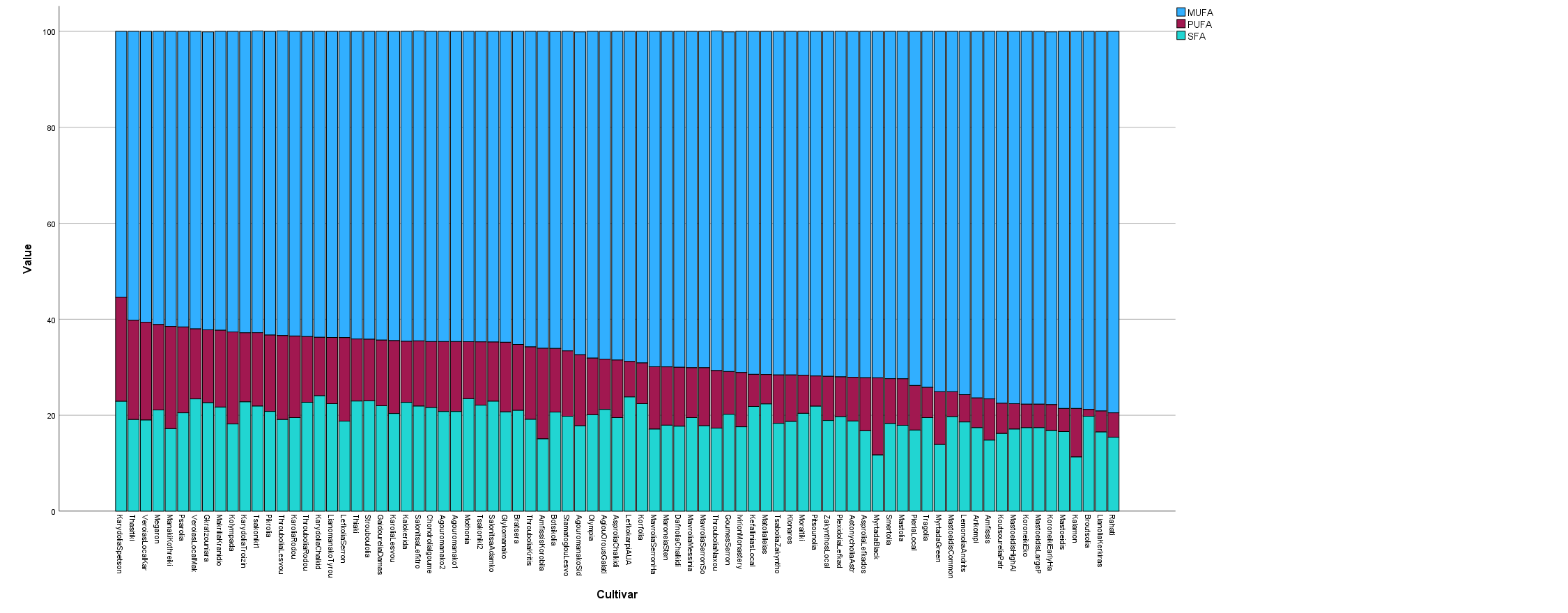


**Supplementary Figure 4. Fatty Acid Composition of Olive Cultivars.**

Bar plot, generated using SPSS software, illustrating the distribution of monounsaturated (MUFA, blue), polyunsaturated (PUFA, red), and saturated fatty acids (SFA, teal) across the studied olive cultivars. Each cultivar is listed alongside its corresponding fatty acid profile, providing a clear visual comparison.
